# Immunotherapy and cancer: the CIPOMO viewpoint on challenges and opportunities

**DOI:** 10.1093/oncolo/oyag435

**Published:** 2026-11-04

**Authors:** Giuseppe Aprile, Donatella Iacono, Sandro Barni, Monica Giordano, Cinzia Ortega, Rosa Rita Silva, Paolo Tralongo, Luisa Fioretto, CIPOMODAY 2024 Scientific Committee, Giuseppe Aprile, Monica Giordano, Cinzia Ortega, Rosa Rita Silva, Giovanni Pietro Ianniello, Katia Cannita, Clementina Savastano, Antonio Iaria, Fabrizio Artioli, Alessandro Follador, Teresa Gamucci, Carlo Aschele, Andrea De Monte, Renato Bisonni, Roberta Buosi, Silvana Leo, Maria Giuseppina Sarobba, Stefano Vitello, Angela Stefania Ribecco, Orazio Caffo, Riccardo Rossetti, Teodoro Sava, Luigi Cavanna, Alberto Scanni, Luisa Fioretto

**Affiliations:** Department of Oncology, Ospedale Universitario Santa Maria della Misericordia, Azienda Sanitaria Universitaria Friuli Centrale (ASUFC), Udine, 33100, Italy; Department of Oncology, Ospedale Universitario Santa Maria della Misericordia, Azienda Sanitaria Universitaria Friuli Centrale (ASUFC), Udine, 33100, Italy; Medical Oncology Unit, Ospedale di Treviglio, ASST Bergamo Ovest, Treviglio, Bergamo, 24047, Italy; Medical Oncology Unit and Oncology Department, Ospedale Sant’Anna, ASST Lariana, San Fermo della Battaglia, Como, 22042, Italy; Medical Oncology Unit, Ospedale “Michele e Pietro Ferrero”, ASL CN2 Alba-Bra, Verduno, Cuneo, 12060, Italy; Medical Oncology Unit, Ospedale di Fabriano, AST Ancona, Fabriano, Ancona, 60044, Italy; Medical Oncology Unit, Oncology Care Network, Azienda Sanitaria Provinciale 8, Syracuse, 96100, Italy; Medical Oncology Unit, Oncology Department, Azienda USL Toscana Centro, Florence, 50012, Italy

## The immunotherapy era and the CIPOMO perspective

In little more than a decade, immune checkpoint inhibitors (ICIs) have advanced from a promising option in advanced melanoma and non–small-cell lung cancer to a backbone of treatment across more than 20 solid-tumor indications.^1–3^ Unlike cytotoxic chemotherapy, ICIs can produce durable and occasionally complete responses, and a meaningful minority of patients with metastatic disease now become long-term survivors.^4^^,^^5^ This success has reshaped the daily work of oncology departments and, with it, the strategic priorities of those who lead them.

The Italian College of the Heads of Hospital Medical Oncology (CIPOMO, Collegio Italiano dei Primari Oncologi Medici Ospedalieri) brings together the directors of medical oncology units across Italy, from large academic centers to community hospitals. From this collective vantage point a consistent set of challenges—and corresponding opportunities—has come into focus. Here we set out what we regard as the defining strategic priorities of the immunotherapy era and the organizational and clinical responses they demand.

## Toxicity is a system-level challenge, not only a clinical one

Although ICIs avoid much of the predictable toxicity of chemotherapy, they introduce immune-related adverse events (irAEs) that are mechanistically distinct, organ-agnostic, and at times life-threatening.^6^^,^^7^ Grade 3-4 events occur in a substantial fraction of patients, particularly with combination regimens, and rare but fatal complications such as myocarditis, encephalitis and pneumonitis demand immediate recognition.^7^^,^^8^ Managing these events is no longer the responsibility of the oncologist alone. It requires standing multidisciplinary networks linking cardiology, neurology, endocrinology, gastroenterology, dermatology and clinical immunology, supported by shared protocols and rapid-access pathways. International guidelines from ESMO and ASCO have done much to standardize care,^9^^,^^10^ but their local implementation—including the education of patients, caregivers and emergency staff who often encounter irAEs first—remains the practical challenge for departments. The decision to rechallenge after a significant irAE is a recurring dilemma that further underscores the need for structured, expert decision-making.^11^

## A new and growing population: the long-term survivor

Perhaps the most profound consequence of immunotherapy is the emergence of a large cohort of long-term survivors of previously fatal metastatic disease.^4^ These patients raise questions for which oncology has limited precedent: how to govern survivorship care over years rather than months, how to manage chronic irAEs that persist long after treatment, and how to preserve quality of life during extended disease control. Chronic toxicities affecting endocrine, rheumatologic, dermatologic and neurologic systems are increasingly recognized, frequently requiring prolonged steroid exposure or permanent treatment discontinuation.^12^ Defining, monitoring and treating these persistent events is an urgent unmet need, and an opportunity to build dedicated survivorship pathways.^13^

## Special populations and the limits of the evidence

The expansion of ICIs into the adjuvant and perioperative setting extends the promise of cure but shifts the risk–benefit balance: serious or chronic toxicity in a potentially cured patient carries a different weight than in advanced disease. Older adults, who constitute much of real-world practice, remain under-represented in pivotal trials, leaving questions about immunosenescence and tolerability inadequately answered.^14^ Patients with preexisting autoimmune disease face a genuine risk of exacerbation, yet clear guidance on which conditions contraindicate treatment is lacking.^15^^,^^16^ Each of these groups would benefit from deliberate inclusion in trials and from tailored, consensus-based recommendations.

## When to stop treatment

The optimal duration of immunotherapy is among the most consequential open questions in the field. Stopping after a fixed period—often 2 years—in patients achieving a complete or major response has been proposed, but is not universally accepted, and the tradeoff between durable benefit, cumulative toxicity and cost remains unresolved.^17^ Reliable biomarkers to guide both initiation and cessation are still lacking; their development would transform patient selection and treatment de-escalation alike.^18^

## Sustainability and the opportunity of innovation

Financial sustainability is a strategic concern at the system level. The cost of ICIs frequently exceeds departmental budgets, and not all agents offer comparable value.^19^ Whether individual agents are themselves interchangeable remains unsettled: anti-PD-1 and anti-PD-L1 antibodies differ in their molecular targets and pharmacological characteristics, and indirect comparisons suggest only modest differences in efficacy and toxicity, yet adequately powered head-to-head trials are almost entirely lacking.^20^ Where therapeutic equivalence within a class is supported by robust evidence, treating agents as interchangeable could increase competition in purchasing and strengthen price negotiation, with potentially substantial savings for health systems. Several innovations offer realistic relief. Subcutaneous formulations of established agents deliver comparable efficacy with administration in minutes rather than an hour, freeing chair time, enabling care models closer to the patient’s home, and—through future biosimilar competition—potentially reducing cost.^21^ Artificial intelligence holds promise for patient selection, response prediction and toxicity anticipation, although the complexity of real-world data and limited clinical validation temper near-term expectations. Such tools should be approached as aids to, not substitutes for, clinical judgment.

Table 1 outlines the key strategic challenges of the immunotherapy era and highlights the corresponding opportunities and potential strategies to address them.

**Table 1.** Strategic challenges of the immunotherapy era and corresponding opportunities.

Strategic challenge	Opportunity/response
**Immune-related adverse events that are organ-agnostic and occasionally fatal**	Standing multidisciplinary irAE networks; shared protocols and rapid-access pathways; education of patients, caregivers and emergency staff
**A growing population of long-term survivors of metastatic disease**	Dedicated survivorship pathways; systematic recognition and management of chronic irAEs
**Under-represented groups: older adults, patients with autoimmune disease, the adjuvant setting**	Deliberate inclusion in trials; consensus guidance on contraindications and risk–benefit
**Uncertain optimal treatment duration**	Biomarker-guided initiation and discontinuation; prospective de-escalation studies
**Financial and organizational sustainability**	Subcutaneous formulations and biosimilars; AI-supported selection.

## Conclusion

Immunotherapy has changed what oncology can offer, and in doing so has changed what oncology departments must become. The challenges we have outlined—system-level toxicity management, survivorship of metastatic disease, under-served populations, treatment duration, and financial sustainability—are not reasons to qualify past success but the agenda for consolidating it. Meeting them will require multidisciplinary networks, continuous professional development, deliberate inclusion of real-world populations in research, and the thoughtful adoption of innovations such as subcutaneous delivery, biomarker-guided selection and, in time, artificial intelligence. As the leaders of Italy’s hospital oncology units, CIPOMO regards these priorities as shared responsibilities of clinicians, institutions, researchers and policymakers alike. The opportunity before us is to ensure that the remarkable benefits of immunotherapy are delivered equitably, safely and sustainably to every patient who may gain from them.
